# Effect of delivery mode on neonatal outcome among preterm infants: an observational study

**DOI:** 10.1007/s00508-016-1150-2

**Published:** 2016-12-21

**Authors:** Iris Holzer, Rainer Lehner, Robin Ristl, Peter W. Husslein, Angelika Berger, Alex Farr

**Affiliations:** 1 Department of Obstetrics and Gynecology, Division of Obstetrics and Feto-maternal Medicine, Medical University of Vienna, Waehringer Guertel 18–20, 1090 Vienna, Austria; 2 Section for Medical Statistics (IMS), Center of Medical Statistics, Informatics and Intelligent Systems, Medical University of Vienna, Vienna, Austria; 3 Department of Pediatrics and Adolescent Medicine, Division of Neonatology, Pediatric Intensive Care and Neuropediatrics, Medical University of Vienna, Vienna, Austria

**Keywords:** Apgar score, Birthweight, Delivery mode, Neonatal outcome, Preterm delivery

## Abstract

**Background:**

The optimal mode of delivery as a predictor for outcomes in preterm infants is under debate. The purpose of this study was to evaluate the effect of the delivery mode on neonatal outcome among preterm infants in different birthweight categories.

**Methods:**

A retrospective analysis of singleton preterm deliveries from 23 + 0 to 33 + 6 gestational weeks was performed. Infants were categorized based on birthweight as large for gestational age (LGA), appropriate for gestational age (AGA) and small for gestational age (SGA). The Apgar score at 5 min served as the main outcome parameter. A sensitivity analysis was performed to adjust for maternal age, parity and fetal malformations as potential confounders.

**Results:**

Out of 1320 singleton preterm infants, 970 (73.5%) were delivered by cesarean section and 350 (26.5%) were delivered vaginally. The AGA infants between 23 + 0 and 27 + 6 weeks showed better outcomes after cesarean section (*p* < 0.01 from 23 + 0–24 + 6; *p* = 0.03 from 25 + 0–27 + 6), whereas AGA infants between 31 + 0 and 33 + 6 gestational weeks showed better outcomes after vaginal delivery (*p* = 0.02). Cesarean section was beneficial in extremely and very preterm SGA infants (*p* = 0.01 from 25 + 0–27 + 6; *p* = 0.02 from 28 + 0–30 + 6). The sensitivity analysis showed no confounding effect of other variables.

**Conclusion:**

There is a benefit from cesarean section in AGA preterm infants until 28 weeks of gestation and in SGA preterm infants until 31 weeks of gestation. Vaginal delivery should be chosen for moderately preterm AGA infants.

## Introduction

Despite the improvement in the prognosis of preterm infants in recent decades, extremely preterm infants still suffer from high morbidity and mortality [1]. In the 1990s cesarean section for deliveries at gestation less than 28 weeks was rarely performed; however, with improving perinatal and neonatal care, cesarean section for preterm infants has become popular due to better postnatal care management for very low birthweight infants. Nevertheless, the optimal mode of delivery in the preterm period continues to be debated by perinatologists. Studies have reported inconsistent results on the relationship between the mode of delivery and neonatal outcomes [2]. Even the latest Cochrane review could not provide clear evidence for the optimal delivery mode among women with preterm labor. Alfirevic et al. [3] reported equal rates of neonatal asphyxia, respiratory distress syndrome, and the Apgar score at 5 min with respect to the delivery mode. In 2006, Muhuri et al. [4] showed that elective cesarean section was associated with decreased infant mortality risks in each very low birthweight category for breech birth infants. For vertex presentation fetuses, the authors proposed a decreased relative mortality risk associated with elective cesarean section, which was significant for those weighing 500–749 g, not significant for those weighing 750–999 g and barely significant for those weighing 1000–1249 g at delivery. In their study, a significantly increased adjusted relative risk associated with elective cesarean section was observed for the 1250–1499 g birthweight group. According to Lee and Gould [5] vaginal delivery was associated with higher mortality rates in a cohort of small for gestational age (SGA) infants at 26–30 gestational weeks. At 31–33 weeks, the current data regarding outcome of vaginally delivered and cesarean delivered SGA infants suggest no statistically significant differences. By contrast, at gestational ages of 34 weeks or more, mortality rates were reported to be significantly lower for SGA infants delivered vaginally [5]. Another study reported that cesarean section even provides survival advantages for the most immature infants who are delivered between 22–25 weeks of gestation, independent of the existing maternal risk factors [6]. Recently published data from the USA showed no improved outcomes after cesarean section, when infants were stratified by the mode of delivery, both in the presence or absence of antenatal corticosteroid administration between 23 + 0 and 36 + 6 gestational weeks [7]. According to this study, respiratory distress syndrome might occur more frequently in infants born by cesarean section in the subgroup with 1500–1999 g.

In contrast to the currently available literature, the aim of the present study was to evaluate the effect of the delivery mode on neonatal outcome among preterm infants, as stratified into different birthweight categories. To our knowledge, no study has yet evaluated this topic.

## Methods

We designed a retrospective data analysis of all singleton preterm deliveries from 23 + 0 (23 weeks plus 0 days) to 33 + 6 (33 weeks plus 6 days) gestational weeks. Eligible deliveries were performed at the Medical University of Vienna, the largest tertiary referral center in Austria, between 1 January 2003 and 31 December 2011. As there was no consensus on the resuscitation of infants before 23 + 0 gestational weeks, only preterm infants ≥23 + 0 (23 weeks plus 0 days) gestational weeks were considered eligible for study inclusion. Multiple pregnancies were excluded from the analysis, as they are known to have an elevated risk for preterm delivery, which again affects neonatal outcome. In order to avoid a potential bias, stillbirths, fetocides and miscarriages were also excluded. All fetuses that were born vaginally were in cephalic presentation. The data were collected based on a cohort of infants that received the current standard of antenatal care at our clinic. Gestational age was routinely assigned either by early fetal ultrasound or the last menstrual period. Cases were identified by the PIA Fetal Database, version 5.6.16.917 for Windows (GE Viewpoint, Munich, Germany). We use standard operating protocols (SOP) for preterm delivery that include the prenatal steroid treatment with betamethasone, given in two dosages of 12 mg each, 24 h apart, as an intramuscular injection. Neonatal outcome was assessed by the Apgar score. As the 5‑min and 10-min Apgar scores usually imply complications of clinical importance, whereas the cause for a low Apgar score at 1 min is often a temporary depression, the 5‑min Apgar score served as a predictor for neonatal outcome. The approach to use the Apgar score at 5 min as our main outcome parameter followed the largest available register study by Thongren-Jerneck and Herbst [8]. Regarding the outcomes of the Apgar score at 5 min, groups with a median score of at least 9 and a 75% quantile of 10 were classified as “good”, where as those with a median of at least 8 were classified as “moderate”, and all other groups with a median below 5 were classified as “bad”.

The vaginal delivery cohort and the cesarean section cohort were divided into four subgroups. The first subgroup included those from 23 + 0 (23 weeks plus 0 days) to 24 + 6 (24 weeks plus 6 days) gestational weeks, the second subgroup those from 25 + 0 (25 weeks plus 0 days) to 27 + 6 (27 weeks plus 6 days) weeks, the third subgroup those from 28 + 0 (28 weeks plus 0 days) to 30 + 6 (30 weeks plus 6 days) weeks and the fourth subgroup those from 31 + 0 (31 weeks plus 0 days) to 33 + 6 (33 weeks plus 6 days) weeks. In the next step, subgroups were again subdivided according to the neonatal birthweight into the following categories: (i) LGA (large for gestational age), (ii) AGA (appropriate for gestational age), and (iii) SGA (large for gestational age). The categories LGA, AGA and SGA were defined by birthweight above the 90th percentile, between the 10th and 90th percentile and below the 10th percentile, respectively.

For each stratum, the Wilcoxon rank-sum test was used to test the null hypothesis of equal distribution of the Apgar score at 5 min between the two groups (vaginal delivery versus cesarean section). Due to the descriptive and explorative nature of the study, no adjustment for multiple testing was performed. The additional univariate and multivariate sensitivity analysis considered maternal age, parity and the presence of fetal malformations as potential confounders that could determine both delivery mode and outcome. For each stratum, a linear model was fit explaining the mean Apgar score at 5 min by the delivery mode and the other variables. The mean value between group differences and 95% confidence intervals calculated from these models were compared to the unadjusted mean differences. Statistical analyses were performed using SPSS Statistics, version 23.0 (IBM, Armonk, NY). The study was approved by the ethics committee of the Medical University of Vienna, and all procedures were performed in accordance with the 1964 Helsinki declaration and its later amendments. Due to the retrospective character of the study, written inform consent was not obtained. Patient records and data were anonymized and de-identified prior to analysis.

## Results

Overall, 1483 cases with preterm delivery between 23 + 0 and 33 + 6 gestational weeks were identified. Out of this cohort, 163 had missing data and were therefore excluded from the analyses. This resulted in a total of 1320 singleton preterm deliveries, which were eligible for study inclusion. Out of this cohort, 970 infants were delivered by cesarean section, whereas 350 were delivered vaginally. The mean birthweight of the infants born between 23 + 0 and 24 + 6 gestational weeks was 623 g. In the group of gestational ages 25 + 0–27 + 6, the mean birthweight was 904 g, and in the group of gestational ages 28 + 0–30 + 6 and 31 + 0–33 + 6 gestational weeks, the mean birthweight was 1348 and 1936 g. Table 1 shows the 10% and 90% quantiles of the observed weight distributions, which were used to classify the infants into the LGA, AGA and SGA categories.

**Table 1:** Quantiles of the observed distribution of infant birthweight in four gestational age categories used to classify infants into LGA, AGA and SGA

Gestational age	10% quantile of birthweight (g)	90% quantile of birthweight (g)
23 + 0 to 24 + 6	492.8	760.0
25 + 0 to 27 + 6	581.6	1150.0
28 + 0 to 30 + 6	826.5	1686.5
31 + 0 to 33 + 6	1288.5	2416.5

We observed the following outcomes with respect to the mode of delivery for infants between 23 + 0 and 24 + 6 gestational weeks: for LGA infants (*n* = 7 in the cesarean section versus *n* = 5 in the vaginal delivery group) outcomes in the study groups were not significantly different (*p* = 0.61). For AGA infants, we observed significantly better outcomes in those that were delivered by cesarean section (*n* = 46 versus *n* = 85; *p* < 0.01). For SGA infants, there was a trend towards better outcomes in the cesarean section group, but the small sample size precluded definite assessment (*n* = 6 versus *n* = 9; *p* = 0.17).

In the group between 25 + 0–27 + 6 gestational weeks, we observed comparable outcomes between the cesarean section and the vaginal delivery group in LGA infants (*n* = 19 versus *n* = 9; *p* = 0.41). For AGA infants, the outcomes of the cesarean section group were significantly better, compared to the vaginal delivery group (*n* = 188 versus *n* = 40; *p* = 0.03). Only 3 SGA infants, born between 25 + 0 and 27 + 6 gestational weeks, were observed in the vaginal delivery group; all of them had a very poor outcome, while those delivered by cesarean section (*n* = 26) had outcomes that were comparable to those of AGA and LGA infants (*p* = 0.01).

In the group between 28 + 0 and 30 + 6 gestational weeks, outcomes of LGA infants that were delivered vaginally were comparable to those delivered by cesarean section (*n* = 11 versus *n* = 27; *p* = 0.15). For AGA infants, we also did not observe a significant difference between the study groups (*n* = 239 versus *n* = 42; *p* = 0.20). The 2 SGA infants of this birthweight category that were born vaginally had an Apgar score at 5 min of 0. The 31 SGA infants delivered by cesarean section had outcomes that were similar to infants of the AGA and LGA category, but significantly different to the SGA infants in the cesarean section group (*p* = 0.02).

In the group between 31 + 0 and 33 + 6 gestational weeks, we did not find a statistically significant difference regarding LGA infants (*n* = 17 versus *n* = 37; *p* = 0.11). For AGA infants, we observed superior outcomes after vaginal delivery, when compared to cesarean section (*n* = 290 in the caesarean section versus *n* = 127 in the vaginal delivery group; *p* = 0.02). We could not test the groups in the SGA category, since there was no SGA infant born vaginally between 31 + 0 and 33 + 6 gestational weeks. Table 2 shows the detailed outcomes of the 1320 preterm infants, including Apgar score, gestational age, birthweight and delivery mode.

**Table 2:** Detailed outcomes of the 1320 preterm infants evaluated by the Apgar score at 5 min in groups stratified by gestational age, birthweight and delivery mode

Gestational age Birthweight	Group	*N*	25% quantile	Mean	Median	75% quantile	*p*-value^*^	Outcome
23 + 0–24 + 6 LGA	C.S.	7 (58%)	7.5	8	8	9	0.61	Moderate
23 + 0–24 + 6 LGA	Vaginal	5 (42%)	5	6.4	8	9		Moderate
23 + 0–24 + 6 AGA	C.S.	46 (35%)	6	6.8	8	8	<0.01	Moderate
23 + 0–24 + 6 AGA	Vaginal	85 (65%)	1	3.5	1	7		Bad
23 + 0–24 + 6 SGA	C.S.	6 (40%)	1.5	4.7	4.5	7.5	0.17	Bad
23 + 0–24 + 6 SGA	Vaginal	9 (60%)	1	2.7	1	6		Bad
25 + 0–27 + 6 LGA	C.S.	19 (68%)	8	7.5	8	9	0.41	Moderate
25 + 0–27 + 6 LGA	Vaginal	9 (32%)	1	6	8	9		Moderate
25 + 0–27 + 6 AGA	C.S.	188 (82%)	8	8.1	8	9	0.03	Moderate
25 + 0–27 + 6 AGA	Vaginal	40 (18%)	7.8	7.5	8	9		Moderate
25 + 0–27 + 6 SGA	C.S.	26 (90%)	7.2	7.8	8	9	<0.01	Moderate
25 + 0–27 + 6 SGA	Vaginal	3 (10%)	0.5	2	1	3		Bad
28 + 0–30 + 6 LGA	C.S.	27 (71%)	4	6.8	8	9	0.15	Moderate
28 + 0–30 + 6 LGA	Vaginal	11 (29%)	8	8.3	9	10		Good
28 + 0–30 + 6 AGA	C.S.	239 (85%)	8	8.3	9	9	0.20	Good
28 + 0–30 + 6 AGA	Vaginal	42 (15%)	8	8.5	9	9		Good
28 + 0–30 + 6 SGA	C.S.	31 (94%)	8	8	8	9	0.02	Moderate
28 + 0–30 + 6 SGA	Vaginal	2 (6%)	0	0	0	0		Bad
31 + 0–33 + 6 LGA	C.S.	37 (69%)	8	8.6	9	9	0.11	Good
31 + 0–33 + 6 LGA	Vaginal	17 (31%)	8	8.5	10	10		Good
31 + 0–33 + 6 AGA	C.S.	290 (70%)	8	8.7	9	10	0.02	Good
31 + 0–33 + 6 AGA	Vaginal	127 (30%)	9	9	9	10		Good
31 + 0–33 + 6 SGA	C.S.	54 (100%)	8	8.6	9	9	n/a	Moderate
31 + 0–33 + 6 SGA	Vaginal	0 (0%)	–	–	–	–		–

*C.S.* cesarean section, *LGA/AGA/SGA* large/appropriate/small for gestational age, *N* number of observations (percentage within the stratum of cesarean section or vaginal delivery mode), *n/a* not available

^*^ *p*-value from a Wilcoxon rank-sum test for comparison of Apgar score at 5 min between the delivery groups

For 151 infants, the Apgar score at 5 min was not reported, and for an additional 12 infants the birthweight was missing. These cases were excluded from the analyses. In 3 other cases, maternal age was missing, so that these cases were excluded from the sensitivity analysis, performed to detect potential confounders. The estimated mean differences remained stable after adjustment for the included variables, supporting the validity of our primary analysis, as there was no effect on the Apgar score at 5 min (Table 3).

**Table 3:** Results of the sensitivity analysis for maternal age, parity and fetal malformation as potential confounders

		Univariate^a^	Multivariate^b^
Gestational age	Birthweight	Mean difference (95% Cl)	Mean difference (95% Cl)
23 + 0–24 + 6	LGA	1.6 (−2.6 to 5.8)	0.1 (−4.3 to 4.6)
23 + 0–24 + 6	AGA	3.3 (2.3 to 4.3)	2.9 (1.8 to 4.1)
23 + 0–24 + 6	SGA	2.0 (−1.9 to 5.9)	1.5 (−1.6 to 4.5)
25 + 0–27 + 6	LGA	1.5 (−1.6 to 4.5)	1.0 (−1.3 to 3.3)
25 + 0–27 + 6	AGA	0.6 (−0.1 to 1.4)	0.3 (−0.2 to 0.9)
25 + 0–27 + 6	SGA	5.8 (−0.4 to 12.0)	5.9 (3.2 to 8.5)
28 + 0–30 + 6	LGA	−1.3 (−3.7 to 1.1)	−0.9 (−3.2 to 1.4)
28 + 0–30 + 6	AGA	−0.2 (−0.8 to 0.3)	−0.2 (−0.7 to 0.3)
28 + 0–30 + 6	SGA	8.0 (7.1 to 8.8)	8.0 (4.6 to 11.5)
31 + 0–33 + 6	LGA	0.1 (−1.4 to 1.6)	−0.3 (−1.4 to 0.8)
31 + 0–33 + 6	AGA	−0.3 (−0.5 to 0.0)	−0.3 (−0.6 to 0.0)
31 + 0–33 + 6	SGA	n/a	n/a

*LGA/AGA/SGA* large/appropriate/small for gestational age, *CI* confidence interval, *n/a* not available

^a^Univariate sensitivity analysis

^b^Multivariate regression model (*p*-values available on request)

## Discussion

The present study aimed to evaluate the effect of the delivery mode on the outcome of preterm infants, determined by the Apgar score at 5 min. Essentially, we found a benefit from cesarean section among AGA infants until 28 weeks of gestation, SGA infants until 31 weeks of gestation, as well as from vaginal delivery among moderately preterm AGA infants.

There have been several attempts to provide evidence with prospective randomized studies evaluating the optimal mode of delivery in preterm infants; however, none of these trials could successfully answer the question due to the major problems of recruiting pregnant women, crossover, and ethical considerations [9]. It is known that the rate of cesarean section is significantly higher in the preterm period, compared to the term period, as there are several indications for preterm cesarean sections. Several criteria must be ensured to enable vaginal delivery for preterm infants. Regular labor contractions, intrapartum fetal cardiotocography without pathological features, and the absence of congenital fetal anomalies necessitating pediatric surgical intervention are required. Other causes that indicate elective cesarean section are maternal comorbidities that do not enable vaginal delivery. Neonatal birthweight, as stratified by the categories LGA, AGA, and SGA, can also be used as an indicator for the comorbidities of infants. Published data suggest that SGA infants are at greater risk for problems manifested during the first year of life [10]. Furthermore, the SGA classification seems to be related to early developmental delays and language problems later in the life of these infants [11]. These sequential problems might be among the causes of the previously rising cesarean section rate in preterm SGA infants from 50% in 1995 to 61% in 2003 [12].

The results of our study lend support to the idea that there might be a benefit for AGA infants born by cesarean section at 23 + 0–27 + 6 gestational weeks. This seems to be inconsistent with the findings of Malloy et al. [13], who reported that cesarean section is neither associated with a lower risk of mortality nor with intraventricular hemorrhage in very low birthweight infants. In addition, the currently available literature suggests that there is no difference regarding potential markers for intrapartal asphyxia and a 5-min Apgar score of less than 7 between both delivery modes [13]. Our data suggest that SGA infants at 25 + 0–30 + 6 gestational weeks show better outcomes when delivered by cesarean section. Deulofeut et al. [9] described a higher risk of periventricular leukomalacia for infants weighing less than 1250 g, as well as a higher risk of severe intraventricular hemorrhage for infants less than 750 g, when delivered vaginally. These findings seem consistent with our results, which indicate superior outcomes for SGA infants delivered by cesarean section until 31 weeks of gestation. In contrast, a large US study on SGA infants between 25 and 34 weeks reported that cesarean section was not associated with improved neonatal outcomes but even higher rates of respiratory distress syndrome and lower Apgar scores [12]. In contrast to this study, which reported a vaginal delivery rate of 41.2% in the SGA cohort, our data on vaginally delivered SGA infants were insufficient to clearly recommend the ideal mode of delivery. Of note, the matter of being SGA (in comparison to AGA) does not seem to be associated with the occurrence of respiratory distress syndrome [14]. The fact that we could not find a significant benefit from a delivery mode in the AGA and LGA category between 28 + 0 and 30 + 6 gestational weeks, appears to be consistent with the currently available literature [7, 13]; however, according to our results, vaginal delivery seems to be beneficial for AGA infants at 31 + 0 and 33 + 6 gestational weeks.

We are aware that our study has several limitations. These include the retrospective nature of the collected data, as well as the fact that we could not differentiate between women who underwent induction of labor and those who began labor spontaneously. In addition, we were not able to distinguish between elective and unplanned cesarean delivery. Since our hospital is the largest nationwide referral center, there are various causes for preterm delivery, including pregnancy-related, non-pregnancy-related, maternal, and fetal complications. In our analysis, we did not differentiate between these causes, so there might have been different indications for the cesarean section. Regarding the 5‑min Apgar score, we could also have used the composite neonatal outcome score or other parameters (e. g., neonatal death prior to discharge, respiratory distress, sepsis, intraventricular hemorrhage, seizure and subdural hemorrhage) as neonatal outcome parameter [7, 15]; however, we considered the Apgar score at 5‑min as the best available parameter, as it is easy to use and well-known for both obstetricians and neonatologists. Moreover, it has already been shown to have adequate applicability in previous studies [12, 13, 15]. We also admit that the LGA and SGA groups were small in comparison to the AGA groups and, therefore, this analysis might lack statistical power. Since our study followed an observational design, the results should only be interpreted within the limits of this approach. Finally, we were unable to rule out a selection bias due to the differences in the distribution of delivery mode between the infants. Indeed, we cannot yet conclude that there is a causal relationship between the mode of delivery and the outcome of the preterm infant.

In conclusion, our data indicate that SGA preterm infants that are delivered vaginally have moderate to bad outcomes, regardless of gestational age. In our study, we found a significant benefit from cesarean section for SGA preterm infants until 31 weeks of gestation. The majority of LGA preterm infants showed good outcomes, regardless of the delivery mode and gestational age. We also observed better outcomes in AGA infants until 28 weeks of gestation that were born by cesarean section, whereas those between 31 + 0 and 33 + 6 weeks had superior outcomes after vaginal delivery. Larger prospective studies are warranted to confirm our findings.
